# Bio-inspired cognitive robotics vs. embodied AI for socially acceptable, civilized robots

**DOI:** 10.3389/frobt.2026.1714310

**Published:** 2026-01-13

**Authors:** Pietro Morasso

**Affiliations:** Italian Institute of Technology, Center for Human Technologies, Robotics, Brain and Cognitive Sciences and COgNiTive Architecture for Collaborative Technologies Research Units, Genoa, Italy

**Keywords:** computational frugality, developmental psychology, ecological psychology, embodied artificial intelligence, embodied cognitive science, enaction theory, prospection, simulation theory of cognition

## Abstract

Although cognitive robotics is still a work in progress, the trend is to “free” robots from the assembly lines of the third industrial revolution and allow them to “enter human society” in large numbers and many forms, as forecasted by Industry 4.0 and beyond. Cognitive robots are expected to be intelligent, designed to learn from experience and adapt to real-world situations rather than being preprogrammed with specific actions for all possible stimuli and environmental conditions. Moreover, such robots are supposed to interact closely with human partners, cooperating with them, and this implies that robot cognition must incorporate, in a deep sense, ethical principles and evolve, in conflict situations, decision-making capabilities that can be perceived as wise. Intelligence (true vs. false), ethics (right vs. wrong), and wisdom (good vs. bad) are interrelated but independent features of human behavior, and a similar framework should also characterize the behavior of cognitive agents integrated in human society. The working hypothesis formulated in this paper is that the propensity to consolidate ethically guided behavior, possibly evolving to some kind of wisdom, is a cognitive architecture based on bio-inspired embodied cognition, educated through development and social interaction. In contrast, the problem with current AI foundation models applied to robotics (EAI) is that, although they can be super-intelligent, they are intrinsically disembodied and ethically agnostic, independent of how much information was absorbed during training. We suggest that the proposed alternative may facilitate social acceptance and thus make such robots *civilized*.

## Introduction

The expected dissemination of cognitive robots in our society at all levels, from industry to the multiform service sector, highlights the importance of focusing on the ethical aspects that may affect their interaction with human users and/or partners. An example is safety in physical interactions: robots are complex moving machines, and their physical interaction with humans implies the danger of fatal accidents. This danger can be attenuated, as in all the electromechanical devices of common use, by strict design/fabrication standards and reactive control mechanisms. However, such an approach only covers the dangers due to physical interactions, not the cognitive interactions: intelligent robots are autonomous agents, whose immediate or final goals may conflict with the intended goals of specific human partners and/or society in general. Science fiction literature masterfully illustrates the insurmountable dilemmas that must be faced by robot designers, as well as regulating institutions to provide a solid and reliable ethical foundation for intelligent humanoid robots operating with a significant degree of autonomy in human society. Consider, for example, the dramatic conflict situations narrated in several robot stories by Isaac Asimov, due to an ethical misalignment between humans and intelligent robots.

This paper will focus on how the outlined ethical dilemmas are dealt with according to two different roadmaps for the development of intelligent robots interacting with humans in a cooperative manner: 1) a roadmap based on large foundation models of AI, specialized for robot behavior (EAI: Embodied Artificial Intelligence) and trained via machine learning; 2) a bio-inspired roadmap based on fully embodied cognition and trained via neurodevelopment and social interaction. Before focusing on such an issue to support the rational choice of one roadmap or the other, let us briefly consider the relationship between intelligence, ethics, and wisdom in general to highlight how such a concept may impact the roadmaps mentioned above.

## Intelligence, ethics, wisdom

Human (as well as animal) intelligence includes a problem-solving ability based on the accumulation of knowledge through learning and experience. It can be “weighted”, i.e., it can grow with the amount of assimilated knowledge, and it can be “tested/measured” by specific protocols. However, intelligence is intrinsically uncommitted to ethical values, similar to the scientific method in general, where *truth* via experimental evidence is the fundamental value, independent of moral considerations, whether the application of scientific discoveries is *right* or *wrong* under current laws.

Wisdom is something else. In contrast with intelligence, it is defined by how knowledge and experience is used for making good decisions, where the distinction between *good* and *bad* is the fundamental value and the ultimate goal is to allow the wise cognitive agent to achieve some personal and/or collective happiness. This kind of formulation was well-presented in classical Greek philosophy, using the term *phronesis* to characterize the type of human knowledge capable of guiding an individual in the selection of the course of action in a moral sense. Moreover, an essential aspect of the peculiar expertise that allows an intelligent agent to achieve wisdom, namely, identifying the intellectual process that underlies wisdom, has emerged from another philosophical tradition, typical of the Eastern mindset. Consider, for example, the work of Herman Hess (1922) in the philosophical novel Siddharta, which is inspired by the Indian intellectual tradition. In the words of Siddharta “*Wisdom cannot be passed on. Wisdom which a wise man tries to pass on to someone always sounds like foolishness … knowledge can be conveyed, but not wisdom. It can be found, it can be lived, it is possible to be carried by it … but it cannot be expressed in words and taught*.” In other words, knowing how to do (i.e., intelligence) and knowing how to choose (i.e., wisdom) are independent although related human features. Anyone needs some intelligence to be wise, but the pure accumulation and assimilation of encyclopedic knowledge does not imply wisdom. There is also experimental evidence, from psychological studies, that the relationships between intelligence and wisdom in individuals is far from linear (Glück and Scherpf, 2022).

As suggested by Siddharta, intelligence can be taught and/or learnt, whereas wisdom cannot. From the individual point of view, wisdom is the ultimate state achieved by full self-consciousness and equilibrium of the mind: it is a deep but elusive concept that was already present in the Greek mindset, when the expression “*Know yourself (gnōthi sautón)*” was carved on the front of the Apollon temple in Delfi, meaning that God urged humans to recognize their own limitations and finiteness as basis of their wisdom. Projecting this human faculty from the personal to the social context we may suggest that wisdom is the essential ingredient to achieve and exert effective leadership. Of course, this is not a linear process, and human history is witness to its ragged evolution, in a competitive/cooperative relationship with the parallel evolution of scientific/technological know-how. In general, we argue that the potentially contradictory relationship between intelligence and wisdom should and could be solved in an ethical framework founded on two principles: a) bidirectional interaction between intelligence and wisdom, avoiding any hierarchical relationship (including the over-reliance on super-intelligence), and b) responsibility and accountability of the decision-making process, taking into account that human wisdom may be affected by several potentially conflicting components (Jeste and Lee, 2019).

The link between AI and intelligence/knowledge is an oxymoron, but what is the possible relationship between AI and wisdom? Although AI as a scientific research area initiated more than 50 years ago, born as a branch of the cybernetic framework defined by Norbert Wiener, the most significant part of the current wave is articulated in large foundation models, based on computational methods not much different from the early neural network models, and is motivated and over-emphasized by the expectation to achieve in a short time AGI (Artificial General Intelligence), i.e., the capacity to replicate and ultimately overcome human-level cognition (Bostrom, 2014; Goertzel, 2014; Martin and Mani, 2024). Whether this expectation stands on solid ground and scientific evidence is still too early to say, and the issue will probably be solved more in economic than scientific terms. Current AI foundation models are far from the minimalistic attitude implied by the so-called *Occam’s razor*: they are massively data and energy-voracious and organized in such a way as to accumulate an endless amount of facts and behaviors, ultimately distilled into an immense and growing body of encyclopedic knowledge. There is no doubt that such models are intelligent, for example, are capable of passing the Turing test, and can beat most human subjects in specific intellectual competitions. However, they are just powerful tools and in no way can represent a new scientific paradigm, an opinion expressed by Xu et al. (2024). Thus, it is pretty natural to anticipate that such AI tools will be employed massively and systematically throughout society and the economic system: powerful new tools to expand intellectual capabilities as the adoption of motors expanded the power capabilities required in agriculture and industry, and the development of ICT (Information and Communication Technologies, including the internet) significantly increased industrial productivity. On the other hand, the growing success of foundation models has also pushed forward the unsubstantiated belief, even in the scientific community, that the massive accumulation of knowledge by itself will ultimately transform AGI into an authoritative autonomous system, independent of human supervision, capable of selecting the best possible choice in any conceivable circumstance. In short, it has been suggested that “*AI is becoming the pinnacle of human wisdom*” (Xu et al., 2024). This expectation is scientifically unsupported, ethically unacceptable, and socially dangerous, although AGI is a formidable problem-solving machine, to be used and trusted with great care.

The supreme danger of attributing wisdom to intelligent autonomous systems, entitling them to select the best course of action in any circumstance involving humans, is well exemplified by two movies by Stanley Kubrick: “*Dr. Strangelove or how I learned to stop worrying and love the bomb*” and “*A space odissey*”. Although aiming at general forms of Artificial Wisdom is a poorly posed and/or dangerous goal, analyzing and evaluating the ethical consequences of a massive dissemination of AGI is a hot issue (European Commission, 2019). The central dilemma (Sternberg and Glück, 2019) is that although wisdom and ethics are closely intertwined, we still lack solid experimental evidence for understanding how individuals, recognized as wise, face moral dilemmas while developing ethical views: ethics is an integral aspect of wisdom, in the sense that metaphorically wisdom may be viewed as an inner spring source which feeds the flow of ethical behaviors. An approach to escape from the paralyzing effect of the wisdom dilemma is formulated by Caniglia et al. (2023) as the concept of “practical wisdom”, i.e., the central virtue of citizens involved in public and social life and called to make decisions when values are conflicting, power is unequal, and knowledge is uncertain. The proposal is to focus on sustainability research by strengthening the capacity to define an appropriate normative framework. This is equivalent to developing a legal/value system to be interrogated/consulted in conflicting situations.

## Embodied cognition as the spring source of ethics and wisdom

The embodied nature of human cognition and intelligence comprises both technological and computational aspects. Neurons and neural networks are the building blocks throughout the whole sensory-motor-cognitive system of humans and the great variety of intelligent animals. Such networks operate in real time with a combination of digital and analog information supported by various electrical and chemical interactions. These networks are trained by interaction with the stimulating environment already before birth and then in a process of neurodevelopment through the maturation stages investigated by Piaget (1952), which support multi-sensory fusion and sensory-motor calibration, the discovery of affordances as described by the ecological psychology of Gibson (1977), the emergence of abstract reasoning and the linguistic characterization of abstract concepts in terms of bodily metaphors (Lakoff and Johnson, 1999), and the powerful amplification effect of social interaction (for example, through education) according to the sociocultural theory of Vygotsky (1978). Human intelligence is produced by this fundamentally embodied and highly personalized process, deeply influenced by specific life experiences, including the awareness of personal limitations and the life/death existential background. In other words, human cognition is intrinsically embodied and ethically sensitive, from birth to death, through the maturation of development and education: this is the fundamental reason for the human propensity to a natural ethical sensitivity and ultimately, if carefully educated and made possible by socio-political institutions, to the acquisition of wisdom. Moreover, the neural computational architecture underlying human embodied cognition is characterized by energetic and computational frugality and surprisingly fast computational cycles, despite the relative slowness of the neural building blocks, as an effect of the generalized parallel processing through the connectome and the mixture of digital and analog computational processes.

In contrast, it is fair to say that the cognitive architectures implied by the foundation models of AI and, in perspective, the ultimate AGI models, are, at the same time, disembodied and ethically-agnostic, by definition and by design. In our opinion, this is a fundamental problem that does not encourage a deep social acceptance and trust but induces a passive and scarcely conscious relationship between humans and intelligent robots. Moreover, in contrast to the computational frugality of human embodied cognition, the AGI model is energy and data-voracious. In the absence of any credible theory of artificial wisdom, it is hard to understand how to modify AI systems, aimed at a fully autonomous AGI, in such a way as to adhere to recognized ethical standards for the application of such systems in real life. The difficulty of such a fundamental ethical problem of AI foundation models has been clearly identified in a white paper by the World Economic Forum (Dignum, 2024), expressing it as the goal of aligning AI systems with human values in general, while recognizing that various stakeholders (e.g., users, regulators, and the public) may express different value systems. The insurmountable obstacle to achieving the goal is that the current AI systems, based on purely computational models, are fundamentally amoral despite their success in several application areas. Thus, any attempt to embed value mechanisms in their structure is complex and unreliable. One idea is to include engineering principles for the ethical alignment of foundation models, for example, formalising the multiple value systems aggregation problem by means of optimisation methods (Lera-Leri et al., 2024) or using utility engineering while tracking the emergence of goals and values during training a foundation model (Mazeika et al., 2025). In any case, science fiction literature illustrates masterfully that such attempts, to provide a solid and reliable ethical foundation for intelligent humanoid robots operating with a significant degree of autonomy in human society, can dramatically fail when unexpected, conflicting, or borderline situations occur: consider, for example, the laws of robotics defined by Isaac Asimov and the multiple situations of conflict narrated in several robot stories.

In general, the main reason why AGI research based on foundation models of intelligence cannot achieve robust levels of ethical alignment is determined by the false, widely held belief that AGI will ultimately replicate and surpass human-level cognition. The main structural difference between AGI and human cognition is that the former is disembodied and amoral by design, whereas the latter is intrinsically embodied and socially sensitive. In other words, it may be suggested that full cognitive embodiment is the *conditio* sine *qua non* which explains the human propensity to ethics (and potentially to wisdom) independent of knowledge and intelligence (Bouhlaoui, 2025; Sandini et al., 2025).

In other words, the design of technical artefacts that imply ethical relevance for their interaction with humans and social matters requires a special and deep focus on both “responsible design” and “responsible use”. In particular, the scarce propensity of foundation models to consolidate an ethical sensitivity can be characterized from two points of view, related to the training and the application phases. In the former case, one may think of inducing ethical propensity by somehow biasing in the ethical sense the training sets employed by machine learning techniques. In the latter case, one may suggest associating a value system with the trained foundation model for ethically biasing the generation of output patterns. However, in both cases, there is no guarantee of the reliability and self-consistency of such computational algorithms if adopted in a completely autonomous manner. In particular, the issue of the definition of value systems, i.e., sets of principles that should guide the development and deployment of AI artifacts and methods, has been faced by various reputable, multi-stakeholder organizations, producing several white papers such as the Asilomar AI Principles^1^ (2017), the Montreal Declaration for Responsible AI^2^ (2017), and the Statement on Artificial Intelligence, Robotics and Autonomous Systems of the European Commission^3^ (2018). Despite the common goal, we are still away from an agreed framework and from identifying an implementation roadmap of such principles. A related dilemma is how to control super-intelligence (Bostrom, 2014), i.e., how human society can remain in control of AI artifacts once a level of super-intelligence has been achieved, without degenerating into choices and behaviors which are not positive according to human perception. The control of super-intelligence is linked to the dilemma for human society about the degree of wisdom that can be attributed to fully or partly autonomous decision making via super-intelligence. According to (Floridi et al., 2018) this is a crucial dilemma that can be formulated as “meta-autonomy”, or a “decide-to delegate” model, based on the principle that any delegation by human society to AGI agents should remain overridable, i.e., the principle of deciding to decide again. According to the authors, the rationale of this principle is to limit the risk of delegating too much to machines, whatever their level of super-intelligence, while protecting the intrinsic value of human choice and the ultimate human responsibility. We also wish to add that, in our opinion, the disembodied nature of super-intelligence determines the rationality of this principle.

The importance of embodiment is clear also to the researchers active in the AI/AGI area who defined a special branch of AI, named EAI (Embodied Artificial Intelligence): the intention is to integrate large foundation models in the cognitive architecture of robotic agents, supporting their ability to perceive, learn from, and interact with a dynamically changing environment, while adapting to the uncertainties of partially or badly specified tasks (Hu et al., 2023). Still, the crucial problem of EAI is that the two subsystems comprising an envisaged AGI robot, namely, the sensory-motor and reasoning-cognitive parts, are so different technologically and computationally that the integration is complex, implausible, and potentially inefficient. A gigantic AGI library to support robot actions may be, at the same time, too much and too little for a humanoid robot operating in cooperation with human or robotic partners. Complex tasks that require careful planning and adaptation to unpredicted and dangerous occurrences can succeed only through teamwork, implemented by a collection of intelligent but specialised and complementary agents, not a collection of equal super intelligent but generalistic replica of the same template, namely, AI artifacts with physical instantiation.

## Feasible roadmap for the development of embodied cognition in robotics

Let us now briefly consider which kind of technology may support the design of fully embodied, bio-inspired robots in a non-distant future. The biological brain, which consists of networks of neurons and glial cells, is capable of parallel and self-organized information processing via biochemical and electrical interactions, with a mixture of digital and analog computation: this computational structure supports adaptation and learning, with a uniform computational architecture throughout the different computational levels (from sensorimotor processing to abstract reasoning). The various attempts to imitate the biological computational paradigm, over more than 40 years, are usually defined as wetware, organic intelligence, etc. (Potter, 2018; Smirnova et al., 2023; Jordan et al., 2024). We may expect that the evolution of this field may follow two roadmaps: 1) to grow artificial brains in the lab out of neurons and glial cells, training them through bidirectional interaction with a complex, structured environment; 2) to synthesize new materials, with chemical-electrical properties similar to the neural tissue and capable to imitate the computational features of the biological neural networks. However, we are still far from a firm scientific and technological grounding for the wetware roadmap.

While waiting for the maturation of the wetware alternative, the most feasible approach that matches most requirements outlined above is neuromorphic engineering and artificial intelligence. Neuromorphic hardware is based on VLSI and associated technologies, which are also the building blocks of the traditional and still predominant digital computers. However, the computing architecture is entirely different in the two cases: the conventional von Neumann architecture is based on the separation between processing and memory, the separation between hardware and software, and the local storage of information; moreover, computation is clock-driven and sequential, with a fully digital organization. In contrast, the neural computation in the brain is asynchronous, parallel, and distributed, integrating computation and storage, without any distinction between hardware and software, and mixing analog and digital signals (Maass, 2016): such features are primarily reflected in the organization of neuromorphic computing despite the technological difference between neuron and neuroglia, on one side, and neuromorphic chips on the other. As a consequence, compared with the von Neumann based AGI framework, which is data and energy voracious, neuromorphic computing can achieve, in principle, a much stronger energy efficiency and a natural learning capability because the neural dynamics, which can unfold on different time scales in different parts of the architecture, is linked to real physical time through the different sensorimotor interaction loops.

In comparison with von Neumann computing, neuromorphic computing is a much younger scientific and technological discipline. Although its rate of development is very high, it is still in a maturation phase where strategic choices still need to be made. However, many neuromorphic chips are already available at different stages of development. Among the distinctive features of neurochips, one is the type of chip (mixed type vs. digital type) and the other is the possibility of on-chip learning (yes vs. no). As an example of the large family of existing chips, we may quote the following ones: 1) BrainScaleS 2 (Grübl et al., 2020), which is mixed analog-digital type with on-chip learning; 2) NeuroGrid (Benjamin, 2014), which is mixed type without on-chip learning; 3) SpiNNaker (Furber et al., 2014), which is digital type with on-chip learning; 4) TrueNorth (Akopyan et al., 2015), which is digital type without on-chip learning. In particular, neuromorphic computing systems have a lot of potential for the development of smart robots based on embodied intelligence and many specific applications of neuromorphic systems to robotics have been reported which demonstrate the superiority of this technology in comparison with more conventional approaches, including AI systems, in terms of power consumption, speed, and accuracy (Aitsam et al., 2022; Sandamirskaya et al., 2022; Putra et al., 2024; Enuganti et al 2025).

However, several challenges tend to slow down the maturation of neuromorphic computing despite its vast potential, particularly in situations where an intelligent agent must interact with a complex physical and social environments, as in robotics (Christensen et al., 2022): for example, the development of scalable methods for sensing, perception, decision making and control, the definition of standard tools for configuring and debugging spiking neural networks, and standards for interfacing neurocomputing architectures to conventional sensors, motors, and computers. Ultimately, the crucial missing element for achieving embodied neuromorphic intelligence (Bartolozzi et al., 2022) will be a better understanding of the algorithmic space that neuromorphic computing substrates enable and facilitate. Similar to biological brains, neuromorphic computing “hardware” drastically differs from the conventional von Neumann architecture, with a substantial equivalence between hardware and software because implementing/programming a neuromorphic algorithm means to design the layout of a neural network architecture with a specific connectivity and specific neural parameters: thus a neuromorphic algorithm is a dynamical system that operates in closed loop with the environment, according to general principles of embodied computation.

A solid general theory for the design of neuromorphic algorithms is not yet available; however, there is ground to believe that such a theory might be guided by the growing understanding of cognitive brain dynamics provided by theoretical brain models that aim to replicate the mind (Grossberg, 2021). Such an evolving theoretical formulation will require large-scale neuromorphic platforms for implementing and testing the feasibility of the approach, for example, BiCoSS (Biological-inspired Cognitive Supercomputing System: Yang et al., 2021), which is a multigranular hybrid non-von Neumann paradigm, based on a scalable hierarchical heterogeneous multicore architecture, capable of implementing multiple computational processes for multiple cognitive tasks in multiple brain areas. We suggest that the algorithmic space made available by BiCoSS or similar computational platforms might encapsulate the cognitive architecture envisaged by Sandini et al. (2025) in the framework of developmental robotics: a roadmap that aims at “baby robots with a minimal set of sensory-motor-cognitive capabilities as the starting point of a training and educational process in close connection with human companions (masters, partners, final users)”.

## Conclusion

We can summarize the main thesis of the paper by saying that the natural propensity of educated humans to develop ethical standards is made possible by embodied cognition, maturated through neuro development and social interaction. On the other hand, all of this cannot be duplicated by machine learning techniques, aimed at the implementation of AGI. Thus, the goal of ethically aligning AGI for empowering it with fully autonomous decision making in critical social matters is not credible and potentially extremely dangerous. Certainly, AGI or successive attempts/implementations for achieving it may well exceed intelligent humans in many competences but human ability to create meaning, make moral choices, and understand things creatively is something AGI can’t imitate. Nevertheless, as the previous industrial revolutions allowed mankind to free itself from physical fatigue, diseases, famine, and organizational stress, by learning to invent, produce and utilize innovative technological tools, we can expect that a similar evolution will occur during the current industrial revolution and the following ones, marked by the advent of AI. In particular, we may expect that the related the scientific and technological transformations that will accompany this process might evolve along two different and somehow complementary roadmaps: 1) Pursuing the current approach to achieve some approximation of AGI, but with a good deal of caveats, i.e., avoiding to over trust its decision-making capabilities but counting on the supervisory capability and responsibility of human teams educated to fully understand the pros and cons of such powerful tool; 2) As suggested by Bouhlaoui (2025), investigating also a different roadmap aimed toward ACI (Artificial Collaborative Intelligence) rather than AGI, i.e., “systems designed to augment rather than replace human cognition while preserving human agency and meaning-making capacity”.

The ACI approach is fully based on the embodiment hypothesis, including the goal of a shared embodied cognition between humans and robots based on a developmental process that, as suggested by Sandini et al. (2025), is the basis for the mutual human-robot understanding, including an ethical framework. Along the same line of reasoning, we may also suggest that as embodied cognition is required for developing a sensitivity to “civilized” ethical standards, it may also facilitate the emergence of a sense of self-consciousness linked, at the same time, to ethics and wisdom.

A suggested roadmap for the development of a computational platform aimed at the integration of embodiment, social acceptance, and neuromorphic computing according to the principles outlined above is the Always-On cognitive architecture (Pasquali et al., 2025) that combines sensor fusion, efficient multimodal in-memory representation of perception, and the self-organization of personal experiences through memory consolidation, in order to enable robots “to continuously perceive and build a self-supervised, emergent representation of the environment to support proactive behavior”. Although there is not yet a neuromorphic implementation of this project, the architecture is already structured as a growing dynamical system, totally alternative to the von Neumann paradigm, namely, a bottom-up approach rooted in minimalism and emergence: rather than imposing predefined cognitive models, the Always-On architecture allows cognition to emerge naturally through continuous, embodied interaction with the physical and social environment.

## Data Availability

The original contributions presented in the study are included in the article/supplementary material, further inquiries can be directed to the corresponding author.

## Footnotes

1 Asilomar AI principles: https://futureoflife.org/ai-principles

2 The Montreal Declaration for Responsible AI (2017): https://www.montrealdeclaration-responsibleai.com/

3 EU statement on Artificial Intelligence, Robotics and Autonomous Systems (2018): https://ec.europ a.eu/info/news/ethics-artificial -intelligence-statement-ege-released-2018-apr-24_en
